# Dehydropyrrolizidine Alkaloid Toxicity, Cytotoxicity, and Carcinogenicity

**DOI:** 10.3390/toxins8120356

**Published:** 2016-11-29

**Authors:** Bryan L. Stegelmeier, Steven M. Colegate, Ammon W. Brown

**Affiliations:** 1 United States Department of Agriculture, Agriculture Research Service, Poisonous Plant Research Laboratory, 1150 East 1400 North, Logan, UT 84341, USA; steven.colegate@usu.edu; 2 Department of Animal, Dairy and Veterinary Sciences, Utah State University, Logan, UT 84322, USA; 3 United States Army Institute of Surgical Research, Ft Sam Houston, TX 78234, USA; brown.ammon@gmail.com

**Keywords:** dehydropyrrolizidine, DHPA, pyrrolizidine, alkaloids, PA, toxic plant, pyrrolizidine alkaloid-induced cytotoxicity, toxic hepatopathy, carcinogenesis

## Abstract

Dehydropyrrolizidine alkaloid (DHPA)-producing plants have a worldwide distribution amongst flowering plants and commonly cause poisoning of livestock, wildlife, and humans. Previous work has produced considerable understanding of DHPA metabolism, toxicity, species susceptibility, conditions, and routes of exposure, and pathogenesis of acute poisoning. Intoxication is generally caused by contaminated grains, feed, flour, and breads that result in acute, high-dose, short-duration poisoning. Acute poisoning produces hepatic necrosis that is usually confirmed histologically, epidemiologically, and chemically. Less is known about chronic poisoning that may result when plant populations are sporadic, used as tisanes or herbal preparations, or when DHPAs contaminate milk, honey, pollen, or other animal-derived products. Such subclinical exposures may contribute to the development of chronic disease in humans or may be cumulative and probably slowly progress until liver failure. Recent work using rodent models suggest increased neoplastic incidence even with very low DHPA doses of short durations. These concerns have moved some governments to prohibit or limit human exposure to DHPAs. The purpose of this review is to summarize some recent DHPA research, including in vitro and in vivo DHPA toxicity and carcinogenicity reports, and the implications of these findings with respect to diagnosis and prognosis for human and animal health.

## 1. Introduction

**Figure 1:**
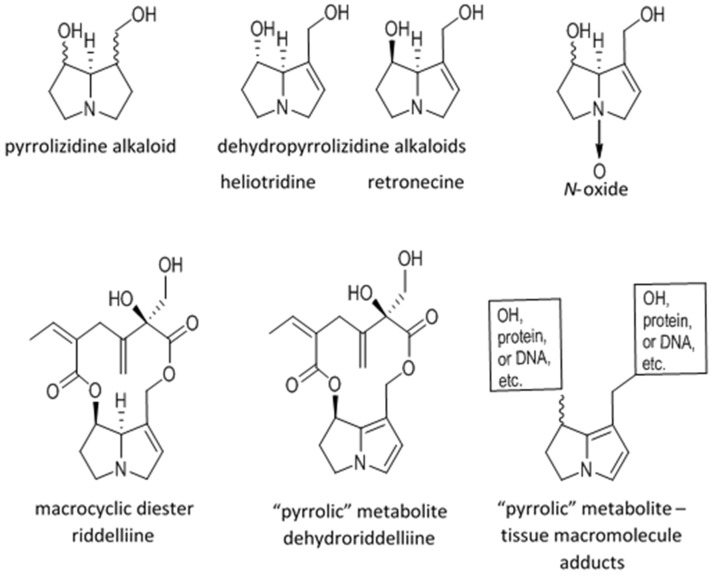
The top row contains the general structure of a non-toxic pyrrolizidine alkaloid. The middle two structures are toxic dehydropyrrolizidine alkaloids, heliotridine and retronecine, with a 1,2 unsaturation that is characteristic of toxic alkaloids. The top right structure is, a dehydroxypyrrolizidine alkaloid *N*-oxide. The second line contains dehydroxypyrrolizidine alkaloid, riddelliine, which is a macrocyclic diester retronecine base alkaloid. The center structure is a “pyrrolic” riddelliine, dehydroriddelliine, or didehydroxypyrrolizidine alkaloid. The last structure on the right is a “pyrrolic” metabolite or macromolecule adduct.

Some plants, including species of *Senecio*, *Heliotropium*, *Echium*, *Symphytum*, *Crotalaria*, *Cyanoglossum*, and *Trichodesma* genera biosynthesize 1,2-dehydropyrrolizidine alkaloids (DHPAs) and have been reported to poison livestock, wildlife, and humans [1,2]. At a fundamental level, pyrrolizidine alkaloids are composed of two fused five-member carbon rings with a bridging nitrogen forming a necine base (Figure 1). The DHPAs have a 1,2 unsaturation that is essential for toxicity (Figure 1). The 1-hydroxymethyl-7-hydroxy substituted necine base is distereoisomeric around C7 and C8 forming either a retronecine or heliotridine base, which are further esterified with a wide range of necic acids at the 1-hydroxymethyl and/or 7-hydroxy substituents forming monoesters, open-chain diesters or macrocyclic diesters (Figure 1). The least toxic have been reported to be the monoesters when compared to more toxic diesters and macrocyclic diesters [1]. The result is that there are more than 350 potentially toxic DHPAs, of which only about 30 have been purified in amounts that have allowed their individual toxicity to be partially characterized. The potential for poisoning, and diagnosis or prediction of poisoning, is further complicated because most plants have mixtures of DHPAs, all of which may be present in the free base or *N*-oxide forms (Figure 1). Although *N*-oxidation is generally considered a metabolic detoxification reaction since it increases solubility and enhances elimination, ingested *N*-oxides can be quickly reduced in the gastrointestinal tract and, under most conditions, their in vivo toxicity is only slightly lower than the free base [3]. 

## 2. Toxicity, Cytotoxicity, and Carcinogenicity

### 2.1. Metabolic Activation and Sequelae

The DHPAs require metabolic oxidation by cellular (most often hepatocyte) cytochrome P450s to their toxic didehydropyrrolizidine derivatives, commonly called pyrroles [1]. These highly reactive, electrophilic pyrroles react with many biomolecules, such as glutathione, which facilitate excretion. The pyrroles can also combine with essential cellular proteins and nucleic acids, forming adducts that may damage cell function and homeostasis. Hepatocytes and endothelial cells are most often affected and this is seen microscopically as degeneration, necrosis, fibrosis, biliary epithelial proliferation, megalocytosis, vascular fibrosis, or veno-occlusive disease and cirrhosis. Some DHPAs also produce extrahepatic damage in the lungs, kidneys, and gastrointestinal tract. 

### 2.2. Conditions of Poisonings

Native DHPA-producing plants are usually not expansive or invasive in distribution and, since they are not very palatable, they rarely poison animals. However, problematic DHPA plants are usually introduced noxious weeds that grow along fences, ditches, and roads where they can expand and invade into adjacent fields and pastures. In some conditions they may dominate plant communities and displace good forages. Livestock poisoning commonly occurs when animals are forced to graze DHPA-producing plants due to the lack of alternative forages or when they eat contaminated prepared feeds. As a general observation, when toxic plants, including DHPA-producing plants, are co-harvested and fed in hay, most animals readily consume them, especially when herdmates compete for the daily ration [4]. Feed contamination does not require large plant fragments as contaminants. Inadvertent co-harvesting DHPA-producing plants with desired teas, grain, or forage has been shown to contaminate the desired product with the DHPAs [5,6,7]. Furthermore, it has been shown that sorghum grain for pig feed, heavily contaminated with DHPA-producing plant parts, still retains toxic levels of DHPAs even after the feed grain has been “cleaned” of all contaminating plant parts and foreign seeds [5]. 

Human poisonings by DHPAs may be more direct. In some areas, DHPA-producing plants have been eaten or used as herbal remedies for generations. Such DHPA use continues and it has been suggested that use is increasing [8]. Alternatively, some herbal products or remedies may be contaminated with DHPAs. Their use has increased and herbal and other traditional medicines can be found in common retail outlets. In the United States alone, the American Herbal Products Association estimates there are over 3000 plant species used in nearly 50,000 herbal products [9]. Most of these products are not regulated and many, inadvertently or intentionally, contain DHPA-producing plants [10]. Some traditional Chinese medicines and other herbal teas sourced in Ireland have been found to contain DHPAs, even though DHPA-containing plants are not listed as ingredients [11]. Similar problems are likely elsewhere. Ignorance of potential DHPA contamination and their potential ill effects are likely to result in their continued use. An example of the ready internet availability of such products is asmachilca tea, which is advertised as being good for bronchial problems in children. This is despite the presence of DHPAs in the source plants and the fact that the young are more susceptible to the adverse effects of the DHPAs [12]. In humans, acute poisoning by DHPAs often produces epidemic-like disease. For example, thousands were poisoned in Afghanistan and Tajikistan as a result of contamination of grain and subsequent flour with co-harvested DHPA-producing plants. These severe diseases are easily identified since they generally result in characteristic clinical signs and lesions [13,14]. Further confirmation can be obtained by indirectly detecting DHPA-related tissue adducts, by chemically cleaving the pyrrole entity from the tissue adduct, trapping as a diether and analysis, for example, using GC/MS, [15,16]. Low-dose or intermittent DHPA exposures that produce subclinical or chronic disease are more difficult to identify. Such exposures are probably more widespread than the acute cases of DHPA intoxications since they include those caused by contaminated natural products, such as honey, pollen, milk, and animal tissues, or when DHPA-containing tisanes, medicinal products, or herbal remedies are used intermittently. These low-level, long-term, or intermittent exposures are likely to produce subtle lesions and very low pyrrole-tissue adduct concentrations. This makes it difficult to link the associated disease and lesions to the DHPAs and obtain a definitive diagnosis (B.L. Stegelmeier, unpublished clinical observations). Preliminary studies of the effects of these low-dose exposures indicate they may be cumulative and such exposure may increase the incidence of neoplastic transformation [17,18,19]. This raises a public health concerns, especially for highly-susceptible populations (fetuses, neonates, etc., as discussed later). 

### 2.3. Toxicokinetics

Data are available for several purified DHPAs including riddelliine, senecionine, monocrotaline, and indicine *N*-oxide [20,21,22,23]. However, only a few of these studies were done using oral exposures which limits extrapolation to clinical disease due to the lack of absorption information. Nonetheless, despite minor differences between alkaloids, about 80% of ingested DHPAs were excreted unchanged in the urine and feces. Using ^14^C-labeled monocrotaline, it was shown that a smaller fraction of about 10% is metabolized and that the ^14^C-labeled carbons are exhaled as CO_2_. An even smaller fraction was detected in the milk. As labeled carbons approached background concentrations in different tissues there is a small DHPA fraction that is lost [21]. This “missing fraction” probably includes DHPAs that are activated, bound to cellular components, or retained as cellular adducts. This hypothesis is supported by recent analyses, including ^32^P-post labeling HPLC and HPLC-esiMS/MS, have made detection of these DNA adducts very sensitive and such adducts have been identified in tissues and DHPA-induced neoplasms years after exposure [24,25]. With time nucleic acids, proteins, and glycolipids containing DHPA-derived adducts are metabolized and repaired. Consequently DHPA-derived adducts are cleared at a low rate. As the adducted “pyrroles” are removed from cellular proteins or nucleic acids it may be that they retain their electrophilicity and again react with cellular components. The progression of chronic disease in animals may help to understand the pathogenesis. Previously-exposed animals often seem to be clinically normal for months and even years before they develop liver disease. This supports the hypothesis that cellular damage continues as adducted “pyrrolic” metabolites are recycled [2]. More research is needed to monitor intracellular DHPA-derived adducts on a molecular basis and better identify the role these adducts play in disease progression. Understanding this process is critical before predicting the risk of such low dose exposures will be possible.

### 2.4. Clinical, Pathological, and Histological Signs of Poisoning

#### 2.4.1. Livestock

**Figure 2:**
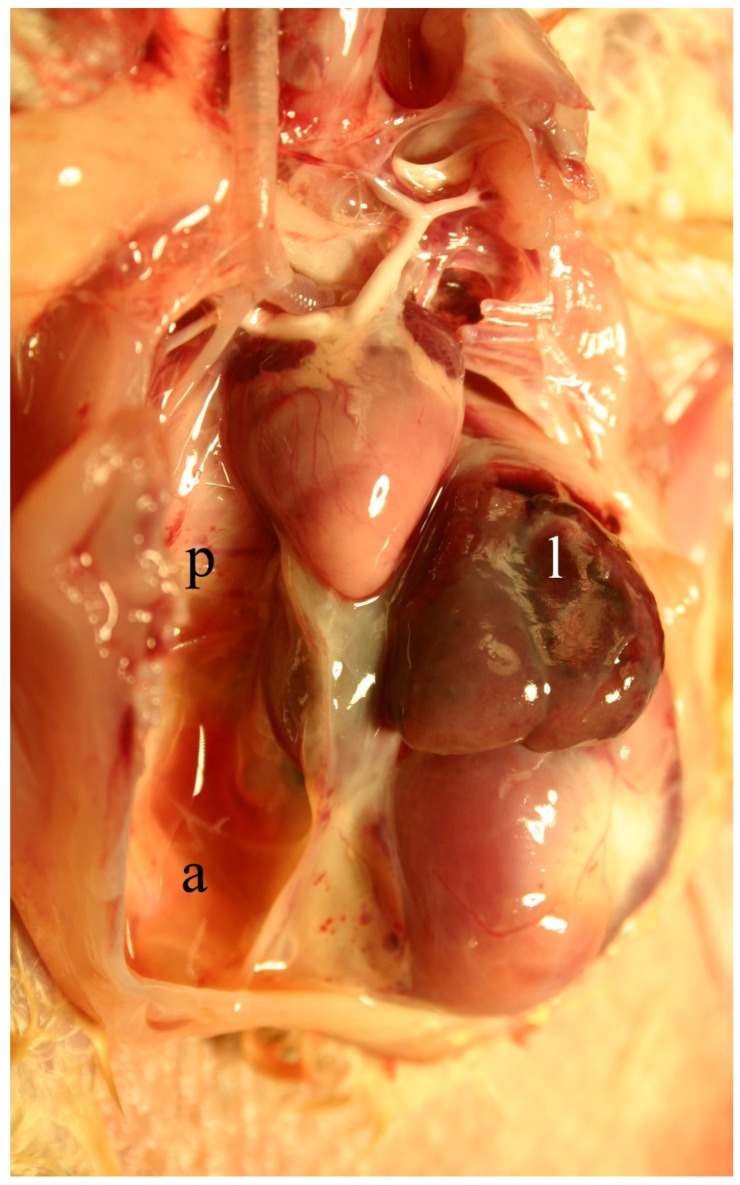
California White chick dosed with riddelliine at 0.26 mMol/kg BW/day for 10 days. Notice the extensive ascites (**a**) with mild fibrinous pleuritis (**p**) and the extremely small and firm liver (**l**).

**Figure 3:**
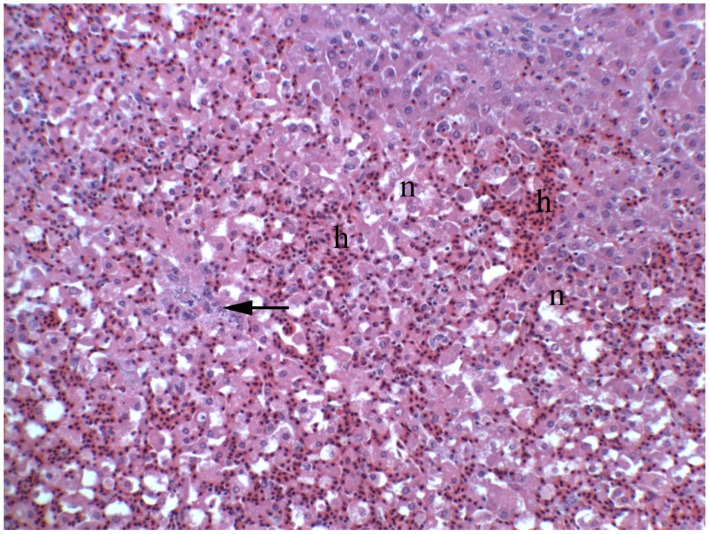
Photomicrograph of the liver from a California White chick dosed with riddelliine at 0.26 mMol/kg BW/day for 10 days. Notice the massive hepatocellular necrosis (**n**) with the collapse of hepatic cords with hemorrhage (**h**). There is minimal periportal inflammation, edema, and early proliferation of ovalocytes (**arrow**).

Acutely intoxicated animals show signs of liver failure, including anorexia, depression, icterus, visceral edema, and ascites (Figure 2). Clinical pathological changes include massive elevations in activity of serum enzymes (AST, SDH, ALK, and GGT) and increased amounts of serum bilirubin and bile acids. Gross and histologic changes often includes panlobular hepatocellular necrosis accompanied by hemorrhage with minimal inflammation (Figure 3). Although some degenerative hepatocytes are enlarged and swollen, and others may fuse forming syncytia. These large cells are not megalocytes, which are characteristic of DHPA-related intoxication, that result from nucleic acid damage and cross-linking with consequent altered cellular mitosis and cytokinesis. 

**Figure 4:**
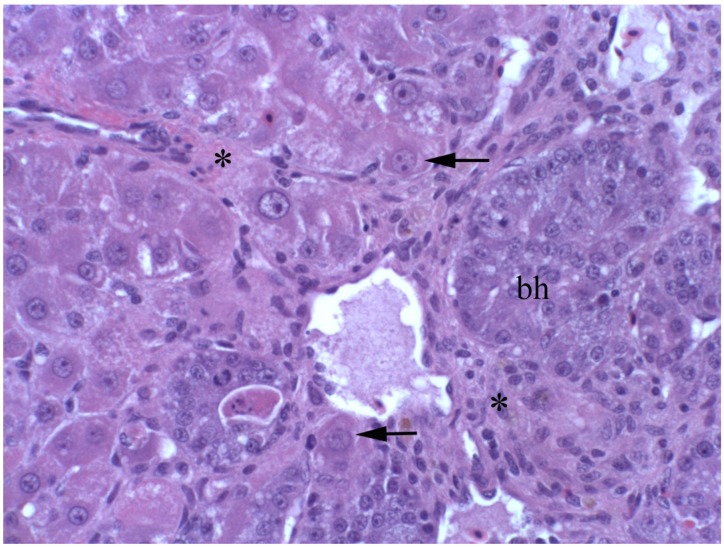
Photomicrograph of the liver of a California White chick dosed with riddelliine at 0.4 mMol/kg BW/ for 10 days. Notice the enlarged hepatocytes (**arrow**) with large nuclei and abundant heterochromatin (most likely developing megalocytes). There is also periportal inflammation and fibrosis (*) with oval cell and biliary epithelial proliferation (**bh**).

Chronic poisoning may not be immediately apparent, clinically, since animals may only develop transient elevations in serum enzyme activities (AST, SDH, ALK, and GGT) and mild elevations in serum bilirubin and bile acids. Hepatic biopsies may be normal or show minimal focal hepatocyte necrosis (piecemeal necrosis), minimal portal and biliary fibrosis, mild bile duct proliferation and, occasionally, megalocytosis (Figure 4). Despite the initial absence of clinical signs, it can be speculated that hepatocellular damage may be progressive as damage continues with focal hepatocyte necrosis and subsequent inflammation, fibrosis, and ultimately cirrhosis. With the resultant loss of hepatic function, animals develop liver failure when they are unable to compensate when stressed with seasonal poor nutrition, pregnancy, or lactation. Such failure may present as photosensitivity, icterus, or increased susceptibility to other hepatic diseases, such as lipidosis or ketosis, with common stresses of seasonal poor nutrition, pregnancy, or lactation. 

There are marked species, individual, age, and gender variations in susceptibility to DHPA-related poisoning. Differences in bioavailability and bioactivation of the DHPAs, and the stability and relative reactivity of the resulting pyrrole all contribute to these variations [1,2]. Highly susceptible species include pigs and chickens, with cattle, horses, and rats being moderately susceptible, and mice, sheep, and goats being relatively resistant [26]. 

#### 2.4.2. Humans

Dehydropyrrolizidine alkaloid-related poisoning in humans is similar to the animal disease with some variations. In acute poisoning, as occurs when grain and flour are contaminated with DHPAs, the lesions include the characteristic necrosis, fibrosis, and biliary hyperplasia. However, these may be overshadowed by vascular damage and fibrosis. The hepatic sinusoids and central veins are commonly affected as they become fibrotic and thickened until they are nearly occluded. This causes increased pressure in the portal venous system with visceral congestion, edema, and extensive abdominal effusions. These changes have been collectively termed hepatic sinusoidal obstruction syndrome or veno-occlusive disease (VOD). However, in some cases, and even within different liver sections, the DHPA-induced VOD may be obscured by end-stage liver disease and cirrhosis. Fetuses, neonates, and young animals are also highly susceptible and there are several animal and human examples of transplacental and transmammary poisoning when fetuses and nursing neonates develop fatal hepatic disease while the pregnant or lactating mothers are unaffected [27,28,29]. 

Chronic poisoning is similar to the animal disease in that it is also more difficult to identify. Chemical extraction, enrichment, and isolation of DHPA-derived adducts is possible but the significance is unknown because they have not been directly linked with disease. Similar situations occur in animals since liver-bound pyrroles in clinically- and histologically-normal cattle and wildlife have been detected (B.L. Stegelmeier, unpublished clinical observations). Since the presence of DHPA derived, tissue-bound adducts are not necessarily linked with loss of function or disease, such findings can be interpreted only to confirm exposure to DHPA-producing plants. As many aspects of poisoning in humans are similar to those of animals, it has been concluded that animal models should be useful in understanding, modeling, and predicting risk or DHPA-induced disease in man [18].

### 2.5. Diagnosis

As many of the clinical and histologic changes of DHPA-related poisoning could be produced by other toxic and infectious diseases, obtaining a definitive diagnosis can be difficult. Various methods have been developed to identify the tissue-bound “pyrrolic” DHPA derivatives but, until recently, none of these techniques have been quantitative [30]. Therefore, interpretation can be difficult and, in many cases, positive results could be interpreted only as evidence of exposure. A recent development using LC/MS detection of the Ehrlich-derivative of the “pyrrolic” diether derived from the tissue DHPA adducts has resulted in more robust quantitation [31,32]. This has been useful in identifying poisoned animals and confirming exposure in cases that may not have produced disease (B.L. Stegelmeier, unpublished clinical observations). Additional developmental work is needed, especially correlating these results with DHPA dose and dose response, to allow predicting the sequelae of poisoning events of varying severity. 

### 2.6. Assessment of Safe Levels

As a result of the health hazards posed by DHPAs, several governmental health organizations and food and drug safety agencies have developed regulations and recommendations limiting the sale and use of products that contain DHPAs, either as natural components or as contaminants. The United States, United Kingdom, Germany, European Food Safety Authority, Dutch National Institute for Public Health, World Health Organization, and Food Standards Australia New Zealand all have limited DHPA exposure or banned certain DHPA-producing plants from herbal products [33]. 

Several DHPAs have been shown to be carcinogenic and genotoxic. However, only riddelliine has been classified as a potential human carcinogen in the United States. This was largely due to a National Toxicology Study where mice and rats were orally dosed with riddelliine (up to 1 mg/kg/day) five days a week for up to two years. About 80% developed hepatic hemangiosarcomas and the incidences of both hepatocellular adenomas and mononuclear cell leukemia were increased [34]. The riddelliine study was possible largely because *Senecio riddellii* (Riddell’s ragwort) can contain nearly 18% riddelliine and practically no other DHPAs [35], thereby allowing access to larger quantities of the relatively pure alkaloid. The concentrations of other DHPAs in other plants rarely exceed 2% or 3% and they are more usually present as mixtures of related DHPAs requiring potentially difficult isolation and purification. Therefore, testing other purified DHPAs using similar studies would be difficult. However, there are several alkaloids that are more toxic than riddelliine and some have been linked to DHPA-induced neoplasms [32,36]. Sensitive models and bioassays that use only milligram quantities of purified DHPAs would be invaluable in evaluating toxicity and carcinogenic potential. With this intention, several models, including two in vitro cellular models, a sensitive in vivo chick model, and a p53 knockout model of carcinogenicity, have been investigated and show promise in providing the ability to determine which alkaloids are likely to be of concern.

#### 2.6.1. Chicken Hepatocarcinoma Cytotoxicity

**Figure 5:**
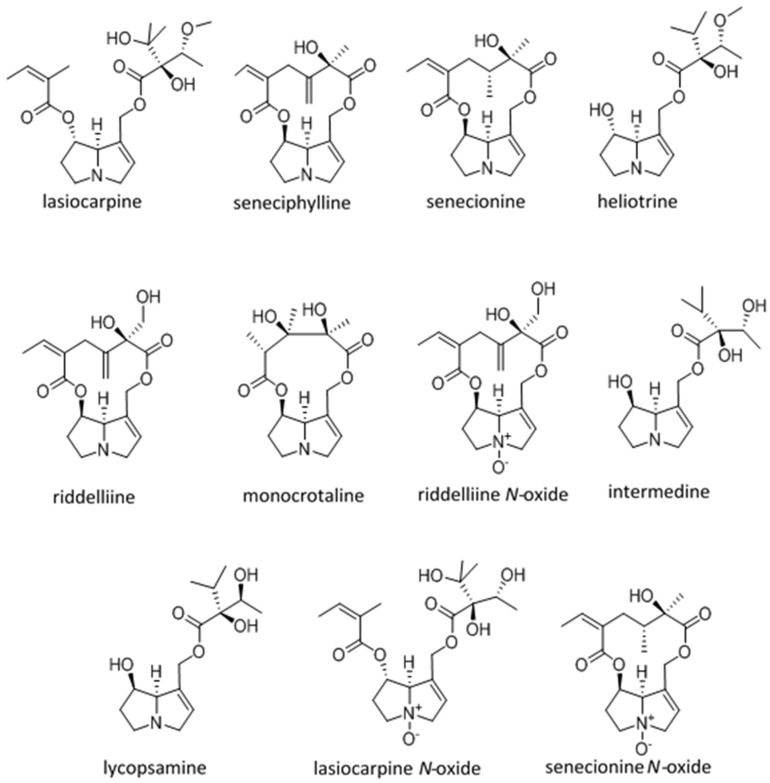
Structures of dehydropyrrolizidine alkaloids that were ranked in this review. Lasiocarpine (heliotridine diester), seneciphylline and senecionine (retronecine macrocyclic diester), heliotrine (heliotridine monoester), riddelliine, monocrotaline, riddelliine *N-*oxide (retronecine macrocyclic diesters), intermedine, lycopsamine, lycopsamine *N-*oxide (heliotridine monoester), and senecionine *N-*oxide (retronecine macrocyclic diester).

After testing several immortalized cell lines, a model of CRL-2118 chicken hepatocyte cytotoxicity was further developed since it was the most sensitive to the DHPAs. This model was used to compare equimolar DHPA exposures between 19 and 300 μM. Cytotoxicity was estimated using cytomorphology, cell viability reflected by mitochondrial function (reduction of tetrazolium dye MTT 3-(4,5-dimethylthiazol-2-yl)-2,5-diphenyltetrazolium bromide), and cellular degeneration reflected by media lactate dehydrogenase activities. Eleven purified DHPAs were compared using this model. Lasiocarpine proved to be the most cytotoxic followed by seneciphylline, senecionine, heliotrine, and riddelliine. The other DHPAs i.e., monocrotaline, riddelliine *N*-oxide, lycopsamine, intermedine, lasiocarpine *N*-oxide, and senecionine *N*-oxide were much less toxic with minimal effect on CRL-2118 cells (See Figure 5 for structures). This comparison identified four DHPAs that were more cytotoxic than the reference alkaloid, riddelliine [36]. 

#### 2.6.2. Primary Hepatocyte Cultures

To evaluate any species-dependent effects of some DHPAs, primary hepatocyte cultures from eight-week-old male chicks, mice, and rats were developed using modifications of a previously described method [37]. The hepatocytes were allowed to establish for 12 h in a 96-well plate. The cells were then exposed to riddelliine for 24 h in concentrations ranging from 0.1–1.2 mM. After cellular exposure, cytotoxicity was estimated using a mitochondrial function assay (MTT). The results suggested that chick primary hepatocytes are about 600 times more sensitive than previously reported for chicken CRL-2118 cells [36]. Mice and rat primary hepatocytes had moderate sensitivities with IC_50_s of about 100 µM. These preliminary results indicated that primary hepatocyte cultures might be used to compare species sensitivity to DHPAs. They also indicated that primary chick hepatocyte cultures, in particular, might be a sensitive model to compare responses of very small (mg) quantities of purified DHPAs (Stegelmeier et al., manuscript in preparation).

#### 2.6.3. In Vivo Chick Bioassay

Historically, DHPA toxicity has been compared using lethal dose data from a variety of studies using different models, methods of DHPA isolation, purification and analysis, and different routes of exposure (Table 1). Immediately obvious is the variability between studies on the same alkaloid. Additionally, the estimated lethal doses (Table 1) are generally not reflected in the clinical response in livestock species (B.L. Stegelmeier, personal observations). Furthermore, it has been shown that most rodents are relatively resistant to DHPA-related poisoning and this would suggest that they are probably not good models for susceptibility or disease in other species. The objectives of the in vivo chick study were to develop a sensitive, small animal model for DHPA poisoning that could be used to compare the toxicity of smaller quantities of seven structurally-diverse DHPAs and three of their *N*-oxides. California White chicks were dosed orally with 0.01, 0.04, 0.13, or 0.26 mmol of riddelliine, senecionine, seneciphylline, echimidine, lycopsamine, lasiocarpine, heliotrine, riddelliine *N*-oxide, senecionine *N*-oxide, or lasiocarpine *N*-oxide/kg bodyweight for 10 days (see Figure 5 for structures). After seven days of recovery, the chicks were euthanized, necropsied, and tissues examined microscopically. Based on clinical, serum biochemical, and histopathological evaluations, as well as pyrrole tissue adduct accumulation, the DHPAs were grouped in relation to their toxicity (Table 1). In this model heliotrine, lasiocarpine and the three macrocylic diesters (seneciphylline, senecionine and riddelliine) are the most toxic. These are followed by the three *N*-oxides (riddelliine *N*-oxide, senecionine *N*-oxide, and lasiocarpine *N*-oxide). Echimidine and lycopsamine are the least toxic of the compounds tested [32]. This model is sensitive for comparison of DHPA toxicity, and data obtained from this research can be used to correlate previous research with this more biologically-relevant model.

#### 2.6.4. Heterozygous p53 Knockout Mouse

The objective of these studies was to develop and use a sensitive, small animal model that would allow “whole body” evaluation of the carcinogenicity of DHPAs. Ideally, the model would require small amounts of purified DHPAs and relatively short incubation times. In the initial study, male heterozygous p53 knockout mice were administered riddelliine at doses of 0, 5, 15, or 45 mg/kg bodyweight/day by oral gavage for 14 days. These doses were selected since the 45 mg/kg bodyweight dose caused minor clinical disease characterized by temporary weight loss and loss of appetite. A final group of the p53 knockout mice was treated with riddelliine at 1 mg/kg bodyweight/day that was included in pelleted feed for 12 months. After 12 months all groups were euthanized, necropsied and the tissues examined microscopically. Exposure to riddelliine increased the incidence of tumor development in a dose-responsive manner (odds ratio 2.05 and Wald 95% confidence limits between 1.2 and 3.4). The neoplasm type was dependent on dose and duration. Mice dosed for 12 months by dietary exposure developed hepatic hemangiosarcomas which were similar to those reported in the other lifetime rodent riddelliine carcinogenesis studies [34]. Angiectasis (peliosis hepatis) with small foci of endothelial dysplasia (probably preneoplastic lesions) and other previously unreported lesions were also observed. The mice exposed to riddelliine by oral gavage for 14 days had more neoplasms than controls, but the neoplasms were mixtures of many different phenotypes that often did not involve the liver. Even the lowest 5 mg/kg bodyweight dose for 14 days had increased neoplastic incidence. This demonstrates that this heterozygous p53 knockout mouse model might be useful to study DHPA-induced neoplasia and indicated that even short-duration, low-dose exposure might be carcinogenic. Certainly, further investigation is needed to better understand the significance of this work. 

## 3. Discussion

The in vitro and in vivo cytotoxicity and carcinogenicity studies just described have some similarities and differences with previous LD_50_ reports of toxicity (Table 1). The LD_50_ comparisons were made using rodents that are generally resistant to DHPA poisoning and, often, they were done using routes very dissimilar from natural routes. The in vivo chick model is a potentially useful development since it is very sensitive and allows simultaneous comparisons of small amounts of DHPAs using the route and duration similar to those that commonly poison livestock and humans. The reported LD_50_ data (Table 1) indicate senecionine has a mean LD_50_ of about 65 mg/kg in mice, which suggests it is the most toxic DHPA of those studied in any of these models. However, there is enough variation between those studies to suggest that there is probably little difference between it and the other DHPAs in the toxic group i.e., lasiocarpine, seneciphylline, senecionine, and riddelliine. Compare this with heliotrine, which has been reported to have an LD_50_ of about 300 mg/kg. This places it in the “low-toxicity” group. However, heliotrine in the in vivo chick model is the most toxic as it was ranked more toxic when comparing weight change, survival, and both pathology scores of ascites and hepatic necrosis [32]. Heliotrine also ranked higher in the chicken hepatocarcinoma (CRL-2118) cytology study with an IC_50_ of about 73 µM, putting it in the toxic group with lasiocarpine, seneciphylline, and senecionine [36]. Heliotrine is a heliotridine-based monoester for which “accepted” structure/toxicity wisdom suggests should be less toxic than a similar diester heliotridine, lasiocarpine. Why heliotrine is unexpectedly more toxic in these recent models is unclear but the results clearly indicate the importance of these parallel comparisons if data are to be extrapolated to assess risk in humans or other animals. It may be that heliotrine is uniquely toxic in chickens. The preliminary primary hepatocyte studies were encouraging and results suggest they might be useful in defining and, perhaps, understanding species differences in toxicity between DHPAs. 

The in vitro cytotoxicity and in vivo chick studies revealed that there are several DHPAs that are more toxic than riddelliine. These include both heliotridine- and retronecine-based alkaloids that comprise both open-chain diesters, macrocyclic diesters, and monoesters suggesting that, in these models, the necic base and character of the necic acid(s) (i.e., monoester, open-chain diester, or macrocyclic diester) probably have less influence on toxicity than do the alkaloid-specific components and stereochemistry in the side chains [32]. More work is needed to determine if these differences are similar in other doses, durations, and species. 

The in vivo chick model studies indicated that general toxicity was not correlated to “pyrrolic”-DHPA derivative or adduct formation. Riddelliine consistently produced more pyrrolic adducts than any other alkaloid in these studies [31,32]. In some cases it was nearly 10 times higher than alkaloids of similar toxicity. Several DHPAs have been associated with various neoplasms [38]. However, riddelliine has been extensively studied with lifetime studies in several species, extensive characterization and descriptions of the neoplasms and proliferative lesions, and molecular and genomic studies documenting the molecular mechanisms of riddelliine-induced neoplasia [39,40,41,42]. These studies contributed to riddelliine’s classification as a potential human carcinogen [43]. More recently Zhao et al. have shown that dehydroriddelliine preferentially binds at the C9 position of DNA forming epimers of 7-hydroxy-9-(deoxyadenosin-*N*-6-yl) dehydrosupinidine and 7-hydroxy-9-(deoxyguanosin-*N*-2-yl) dehydrosupinidine and that these adducts are responsible for riddelliine carcinogenesis at a molecular level [44]. It may be riddelliine is unique and produces many more adducts than similar DHPAs. Such adducts could damage more nucleic acids resulting in more mutations and increased neoplastic transformation. Certainly more research is needed to determine if riddelliine has similar adduct-producing potential in other species, and then determine if adduct production is related to DHPA-induced carcinogenesis. 

The p53 knockout mouse studies with riddelliine also present several points for discussion or consideration. All of the doses increased neoplastic incidence such that a “no observed effect” level was not determined. Certainly in subsequent studies it would be essential to test lower doses to determine if there is a safe dose. This model seems to be sensitive enough to suggest it will be useful in assessing carcinogenicity of other toxic DHPAs, especially those that are more toxic than riddelliine, have been linked to neoplastic transformation, or produce different types and amounts of pyrrole adducts. 

It has been suggested that human hepatocytes are less susceptible to DHPA-induced cytotoxicity, genotoxicity and, subsequently, carcinogenic transformation. Human hepatocytes are also less likely to develop megalocytosis and this has led some to speculate that human nucleic acid repair mechanisms may make them more resistant to DHPA-induced damage [45,46]. However, this may not alter DHPA associated carcinogenesis since areas with a history of DHPA poisoning due to contaminated food are also reported to have increased the incidence of hepatic neoplasms [46]. Certainly more epidemiologic work is needed to confirm such associations. In many ways the disease in the mouse is similar to human DHPA-related disease. Poisoned mice also rarely develop true megalocytes (many murine hepatocytes are normally large and binucleate). Though they are relatively resistant to DHPA-induced liver disease, they are susceptible to DHPA-induced carcinogenesis [34]. The marked differences in species susceptibility to poisoning emphasizes the importance of using caution in extrapolating risks for poisoning or risks of neoplastic transformation between species. 

## 4. Conclusions

DHPAs pose a risk to livestock, wildlife, and humans, but the extent of this risk is not completely understood. Though acute or relatively high-dose poisoning has been studied extensively, the consequences of low-level, chronic sporadic or intermittent exposure is largely unknown. Using in vitro cytotoxicity assays and a sensitive chick bioassay, 12 DHPAs have been directly compared in a biologically-meaningful manner and further confirmed that these DHPAs have differing toxicity that may not reflect the accepted previously-reported toxicities. Some DHPAs are carcinogenic and a sensitive model of carcinogenicity has been identified that promises to be useful in comparing the carcinogenic potential of relatively small amounts of purified DHPAs. Methodology was also developed to indirectly quantitate DHPA-induced tissue adducts and this has been used to document exposure in clinical poisoning. Quantitation of adducts may also be useful in understanding DHPA-related carcinogenesis. 

## Figures and Tables

**Table 1:** Table of dehydropyrrolizidine alkaloid (DHPA) structures with median cytotoxic dose (CT_50_) in chicken hepatocarcinoma cells [36], chick bioassay survival rank [32], and reported toxicity. Columns with different superscripts ^A–E^ indicate significant (*p* < 0.05) differences. The data were statistically analyzed using Statistical Analysis Software (SAS 9.3 Cary, NC, USA). All CT_50_ values are expressed as means ± standard deviation and Tuckey’s range test was used to separate significantly different means. The DHPAs were ranked. Three groups were statistically identified: the toxic group (lasiocarpine, seneciphylline, senecionine, heliotrine), the intermediate group (riddelliine, monocrotaline, and riddelliine *N*-oxide), and the low-toxicity group (intermedine, lycopsamine, lasiocarpine *N-*oxide, and senecionine *N-*oxide).

Dehydro Pyrrolizidine Alkaloid	CRL-2118 Cytology Mean CT_50_ ± SD MTTuM	Chick Survival Log Rank	Chick Hepatocyte Necrosis (Ranking)	Chick Pyrrole nmol/g/Day	LD_50_ mg/kg Male Rat IP (Unless Otherwise Indicated)
Lasiocarpine Diester heliotridine	1 (31 ± 15 ^A^)	4 (4.8 ^A–C^)	7	2 (3.7 ^A^)	77 [47,48] 72 [49,50] 88 IV Rat [51]
Seneciphylline Macrocyclic retronecine	2 (76 ± 35 ^A^)	2 (5.4 ^A–C^)	5	4 (1.6 ^B^)	77 [47,48]
Senecionine Macrocyclic retronecine	3 (96 ± 35 ^A,B^)	5 (3.6 ^A–C^)	2	5 (1.0 ^B,C^)	50 [47,48] 77 mg/kg [52] 85 [50] 57.3 (171 umol/kg) PO mice [22]
Heliotrine Mono ester heliotridine	4 (73 ± 9 ^A^)	1 (8.7 ^A^)	1	3 (3.4 ^A^)	296 [47,48] 300 [53,54] 5000 heliotine-*N*-oxide IP male rat [54]
Riddelliine Macrocyclic retronecine	5 (162 ± 43 ^B^)	3 (5.1 ^A–D^)	3	1 (11.1 ^D^)	105 IV mouse [50,55] 80 PO male rat [56]
Monocrotaline Macrocyclic retronecine	6 (256 ± 65 ^C^)	NA *	NA *	NA *	71 [57] 109 [47,48] 175 [50,58] 510 PO male rat [56]
Riddelliine *N*-oxide	7 (267 ± 280 ^C^)	7 (−1.3 ^B–D^)	6	8 (0.7 ^E^)	~250 PO male rat estimated from adduct production [59]
Intermedine Mono ester retronecine	8 (>300 ^C^)	NA *	NA *	NA *	1500 [50]
Lycopsamine Mono ester retronecine	9 (>300 ^C^)	9 (−13.6 ^E^)	9	9 (0.004 ^F^)	1500 [50]
Lasiocarpine *N*-oxide	10 (>300 ^C^)	8 (−4.9 ^C,D^)	8	7 (0.8 ^C,E^)	547 [60] ~1100 [61]
Senecionine *N*-oxide	11 (>300 ^C^)	6 (−0.6 ^C,D^)	4	6 (1.2 ^C^)	~2000 [61]

* Not analyzed or included in this comparison.
